# P-308. Understanding Recruitment into a Long-acting Injectable Pre-exposure Prophylaxis Trial: Lessons Learned to Enhance Equity and Optimize Engagement of Underserved Groups

**DOI:** 10.1093/ofid/ofaf695.527

**Published:** 2026-01-11

**Authors:** Judith Ratcliffe, Farzana Kapadia, Brandi Moore, Eunice Casey, Emma Kaplan-Lewis, Kruti Gala, Maria Khan, Sahnah Lim, Jason Felder, Ofole Mgbako, Robert Pitts

**Affiliations:** NYC Health+Hospitals, New York, NY; NYU School of Global Public Health, New York, New York; NYU School of Global Public Health, New York, New York; NYC Health and Hospitals, NY, New York; NYC Health and Hospitals, NY, New York; Health + Hospitals, New York, New York; NYU Grossman School of Medicine, New York, New York; NYU Grossman School of Medicine, New York, New York; NYU Grossman School of Medicine, New York, New York; NYC Health+Hospitals, New York, NY; NYU Langone Health, New York, New York

## Abstract

**Background:**

Inequitable uptake of pre-exposure prophylaxis (PrEP), including long-acting injectable PrEP (LAI-PrEP), persists among Black or Hispanic/Latine cisgender men who have sex with men (BLMSM) and cisgender women (BLCGW), and transgender or non-binary (TGNB) persons. Here we describe recruitment efforts and evaluate their success at optimizing representation of these groups in a LAI-PrEP implementation study.

**Figure 1:**
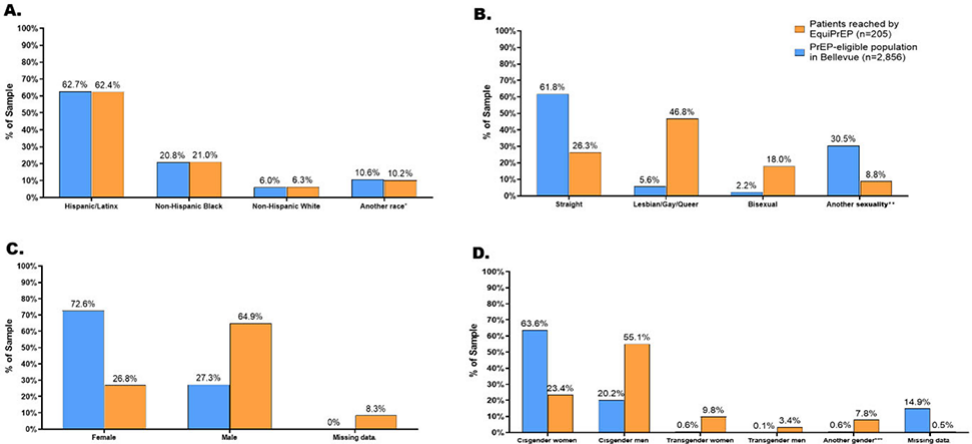
Comparison Patients Eligible for PrEP and Those Recruited for EquiPrEP, an Equity-based, LAI-PrEP Implementation project, NYC, NY, 2023-2024.

**Methods:**

EquiPrEP is an implementation study of uptake and adherence to LAI-PrEP in BLMSM, BLCGW, and TGNB who were >/= 18yo and HIV-seronegative conducted at a municipal hospital in New York City. Hospital-based recruitment and enrollment was conducted between 2/2023-6/2024. Descriptive statistics were employed to compare EquiPrEP recruitment and enrollment to patients who were identified as PrEP-eligible during the same period, using CDC criteria for PrEP eligibility.

**Results:**

During the EquiPrEP study period, n=205 patients were screened for eligibility and n=129 were enrolled with the goal of equally enrolling one-third of participants in each group. A total of n=2856 patients were identified as PrEP-eligible during the same time-period. Comparisons of these eligible to those screened and enrolled in EquiPrEP identified differences by sexual orientation and gender identity. Specifically, participants screened for EquiPrEP consisted of a greater proportion of persons who identified as L/G/B (46.8% vs 5.6%), bisexual (18% vs. 2.2%), cisgender men (55.1% vs. 20.2%), TG (9.8% and 3.4% vs. 0.6% and 0.1%). Further, compared to those eligible (72.6%), only 26.8% of cisgender women were screened for EquiPrEP. Finally, the proportions of patients PrEP eligible compared to those enrolled were comparable across all race/ethnicities. Comparisons between those screened vs. enrolled indicate similar reach.

**Conclusion:**

EquiPrEP enrollment goals for recruitment and enrollment by race/ethnicity were achieved, reflecting local demographic characteristics of this hospital setting. However, additional work to eliminate barriers to enrollment among female/cisgender and TG/NB are still required in order to optimize equitable engagement in LAI-PrEP for all people who can benefit from this HIV prevention strategy.

**Disclosures:**

Emma Kaplan-Lewis, MD, gilead: Grant/Research Support Ofole Mgbako, MD, MS, Gilead Sciences: Advisor/Consultant Robert Pitts, MD MPH, Gilead Inc: Advisor/Consultant|ViiV: Advisor/Consultant

